# Isolation and Characterization of Lytic Bacteriophages from Wastewater with Phage Therapy Potentials Against Gram-Negative Bacteria

**DOI:** 10.5152/eurasianjmed.2022.21010

**Published:** 2022-06-01

**Authors:** Mona Khorshidtalab, İnci Durukan, Enis Fuat Tufekci, Seyran S. Nas, Mujib A. Abdurrahman, Ali O. Kiliç

**Affiliations:** 1 Department of Medical Microbiology, Karadeniz Technical University Faculty of Medicine, Trabzon, Turkey; 2 Department of Medical Microbiology, Kastamonu University Faculty of Medicine, Kastamonu, Turkey; 3 Department of Emergency Medicine, Karadeniz Technical University Faculty of Medicine, Trabzon, Turkey; 4 Department of Biology, Dire Dawa University Faculty of Natural and Computational Science, Dire Dawa, Ethiopia

**Keywords:** Bacteriophages, multidrug resistance, *Myoviridae*, *Podoviridae*, *Siphoviridae*

## Abstract

**Objective:** The increase of multidrug resistance in bacteria has increased the efforts in search of alternative methods. The aim of the present study was to isolate and characterize the lytic phages and assess their lytic activity against a number of gram-negative bacteria.

**Materials and Methods:** The phages and their respective hosts were isolated from wastewater collected from the municipal sewer system of Trabzon, Turkey. The lytic activities of phage were determined using the agar spot test. The identification and antibiotic susceptibility of host bacteria were determined using matrix-assisted laser desorption ionization time-of-flight mass spectrometry and Phoenix 100, respectively. The phages were characterized morphologically using transmission electron microscopy. One of the phages, Enteroc21, which has a broad-host-range, was further characterized by genome restriction endonuclease analysis and burst size.

**Results:** Two phages infected strains of four different species, nine phages were able to infect 2-4 strains belonging to one or two species, and three phages showed lytic activity against only the hosts from which they were isolated. All phages belonged to the *Siphoviridae*, *Myoviridae*, and *Podoviridae* family based on transmission electron microscopy morphology. The Enteroc21 had more than 100 kb genome size and a burst size of 180 per infected cell. Most of the host strains were resistant to ampicillin, amoxicillin–clavulanic acid, and in particular, *Achromobacter xylosoxidans* TRAX 13 was multidrug-resistant showing resistance to cefepime, aztreonam, gentamicin, netilmicin, and ciprofloxacin.

**Conclusion:** This study showed that the isolated phages have the potential to be used in phage therapy against various bacterial infections, including multidrug-resistant bacteria.

**Main Points**

- Fourteen lytic phages and their respective gram-negative hosts were isolated from wastewater.
- One of the host strains identified as *Achromobacter xylosoxidans* was multidrug-resistant showing resistance to cefepime, aztreonam, gentamicin, netilmicin, and ciprofloxacin.
- All isolated phages belonged to the family of *Siphoviridae*, *Myoviridae*, and *Podoviridae* based on their morphological characteristics.
- All phages showed potentials to be used in phage therapy against drug-resistant bacteria.

## Introduction

Bacteriophages (phages) are the most abundant viruses on the earth that infect bacteria. In particular, they are found in the biosphere along with host bacteria. Since the phages are natural enemies of bacteria, they are of great importance in controlling the bacterial population in a wide variety of environments, from wastewater treatment to therapeutic applications.^1,2^

Phages were originally discovered by Felix d'Herelle in 1917, although they were defined by independent researchers Hankin, Gamaleya, and Twort as having structures with antibacterial activity in 1896, 1898, and 1915, respectively. Once the antibacterial activities of phages have been identified, their potential to be used as antibacterial agents in the treatment of bacterial infections has brought out great excitement in the scientific world. With the discovery of antibiotics, the use of phage therapy has reduced significantly and replaced by antibiotics, which had a broad-spectrum activity, and they could be produced to a certain standard.^3^ In addition, biogeographical differences could adversely affect the antibacterial activity of phages. The antibacterial effect of a phage isolated from one geographical region might be ineffective against another strain of the same species isolated from another geographical region.^4^ For these reasons, phage therapy has lost its importance to a great extent after the first half of the 20th century. However, the significant increase in multidrug resistance (MDR) and the ineffectiveness of currently used antibiotics to combat infectious diseases have increased the efforts in search of alternative methods. According to the World Health Organization, antibiotic resistance is one of the most alarming threats to global health. Infections caused by MDR pathogens cause an increase in the treatment costs and the duration of hospital stays of patients.^5^ Therefore, new antibiotics have to be discovered to prevent the development and spread of resistance. Nevertheless, even if new antibiotics have been discovered today, resistance development among bacteria may not be completely prevented due to the selective pressure of antibiotics.^6^

The increasing widespread of MDR among pathogens has made phage therapy one of the important alternatives in the treatment of infectious diseases. Particularly, phages with a broad-host-range and lytic activity will strengthen the treatment options in combating infectious diseases.^7^ The present study aimed to isolate lytic phages infecting gram-negative bacteria from wastewater in Trabzon province, Turkey, and further characterize one of the phages with broad-host-range, Enteroc21, by genome restriction endonuclease analysis and burst size. The results of this study are expected to serve as the basis for new *in vitro* and *in vivo* studies for phage therapy.

## Materials and Methods

### Sample Collection, Bacterial Isolation, and Preparation of Phage Lysate

Two wastewater samples of about one L were collected from two locations of the municipal sewer system of Trabzon, Turkey. For primary isolation of gram-negative bacteria, the wastewater samples were diluted and plated on eosin methylene blue agar, tryptic soy agar, and plate count agar (Lab M, Lancashire, UK). The cultures were incubated at 37°C for 24-48 hours. The bacterial colonies with distinct morphology were purified, gram stained, and used as hosts for lytic phage isolation. The same wastewaters were used for the isolation of lytic phages using the culture-enrichment method described by Lin et al^8^ with some modifications. One hundred twenty-five milliliters of each wastewater was mixed with 125 mL of double-concentrated Luria-Bertani (LB) broth (Lab M) and incubated at 37°C for 24 hours with shaking at 150 rpm. The cultures were centrifuged at 8000 ×g for 10 minutes at 4°C, and the supernatants were filtered using 0.22 μm pore size filters. The phage lysates were stored at 4°C in the dark with one drop of chloroform until use.

### Phage Isolation and Host Range Determination

For the primary isolation of lytic phages, the isolated bacteria were used as hosts by the agar spot test method. Briefly, each host strain was grown in LB broth up to optical density (OD)_600__nm_ of 0.4, and 100 µL of culture was mixed with 5 mL molten soft LB agar (0.6% w/v) at 49°C and poured over the LB agar plates. After the agar solidified and dried, 5 µL of phage lysate was spotted on the agar surface to determine the presence of clear or turbid clear zones in the spot sites. The lysates were then serially diluted in suspension medium (SM) buffer (50 mM Tris-HCl pH 7.5, 8 mM MgSO_4_·7H_2_O, 100 mM NaCl, and 0.002% gelatin) and used to infect phage-susceptible hosts using the double-agar layer method as described above to obtain single phage plaques. The single phage plaque was removed using a sterile pipette tip and resuspended in SM buffer for further purification as described by Lin et al.^8^ The high titer phage lysates were prepared from several soft agar plates. The soft agar with phage plaques was covered with 5 mL SM buffer and incubated for 30 minutes at room temperature on a rotator to release the phage particles in the SM buffer. The soft agar and the buffer were collected in a centrifuge tube using a sterile spreader. The phage particles were separated from the agar and host cell debris by centrifugation at 13000× *g* for 15 minutes at 4°C. The phage titer was determined and used for host range determination using the agar spot test described above. Phages showing lytic activity in more than one strain were identified as phages having a broad-host-range.^9^

### Identification and Antibiotic Susceptibility of Host Strains

The identification of phage-susceptible bacteria was done using standard bacteriological methods and the matrix-assis**t**ed laser desorption/ionization-time of flight mass spectrometry (Microflex, Bruker Daltonik, Bremen, Germany) system. The antibiotic susceptibilities of the strains were determined using the Phoenix 100 system (Becton Dickinson, Sparks, Md, USA). The results were categorized as susceptible, resistant, and intermediate susceptible according to the European Committee on Antimicrobial Susceptibility Testing (EUCAST) standards.^10^ Bacteria resistant to at least one agent in three or more antibiotic classes have been identified as MDR.^11^

### Electron Microscopy of Phages

The morphology of the phages was determined using transmission electron microscope (TEM) using the method of Jamal et al^12^ with minor modifications. Briefly, 5 µL of phage samples (10^10^-10^11^ PFU/mL) were deposited on a formvar-carbon coated 100 mesh copper grids (Electron Microscopy Sciences, Hatfield, Pa, USA) for 2 minutes then washed twice with 5 µL ultra-pure water for one minute. The slide was negatively stained using 2% uranyl acetate (pH 4.5) for 3 minutes and allowed to air dry. The grids were analyzed under a JEM-1010 (JEOL Ltd., Tokyo, Japan) TEM operated at 60 and 80 kV.

### One-Step Growth Curve

The mid-log (OD_600nm_ of 0.4) culture of *Enterobacter cloacae* TRENC 21, the primary host strain of phage Enteroc21, was infected with phage Enteroc21 at an MOI (multiplicity of infection) of 0.1 and incubated at 37°C for 15 minutes for phage adsorption. The infected cells were gathered by centrifugation at 8000× ***g*** for 5 minutes and resuspended in 10 mL Mueller-Hinton broth (MHB, Lab M) and incubated at 37°C with shaking at 180 rpm to allow the phage multiplication. At 15 minutes intervals, 100 μL of phage infected cells were taken for up to 90 minutes and the phage titer was determined. The plaques formed at each time point were used to determine the burst size of the phages by dividing the phage titer at the plateau phase by the initial phage titers.^13^ These experiments were repeated at least three times.

### Phage DNA Isolation and Restriction Endonuclease Analysis

The phage DNA was isolated using standard phenol-chloroform extraction with ethanol precipitation method as described by Sambrook et al.^14^ Briefly, 500 μL of phage lysate of about 10^9^ PFU/mL were treated with 10 µg/mL of RNase and DNase (Sigma-Aldrich, St Louis, Mo, USA) to remove host nucleic acids. Then, the lysate was extracted with an equal volume of phenol–chloroform–isoamyl alcohol (25 : 24 : 1) followed by chloroform-isoamyl alcohol (24 : 1) extraction. The DNA was precipitated with two volumes of 96% ethanol, washed with 70% ethanol, and suspended in 200 μL in Tris-EDTA (TE) buffer (10 mM Tris-1 mM EDTA, pH 7.5). The phage DNA was treated with restriction enzyme digestion using the manufacturer’s instructions and subjected to electrophoresis on a 0.8% agarose gel at 40 V for 6 hours. The gel was stained with ethidium bromide and illustrated under ultraviolet light in a gel documentation system. The genome size of Enteroc21 was determined using *Hin*dIII and *Bst*EII-restricted lambda DNA (NEB, Ipswich, Mass, USA) and GelPilot 1 kb plus ladder (Qiagen, Valencia, Calif, USA) as DNA markers.

### Statistical Analysis

Data were analyzed by calculating mean ± standard deviation, and statistical analysis was performed using Excel 2016 for Windows.

## Results

### Identification of Lytic Phages and Their Primary Host Bacteria

**Figure 1. a,b.**
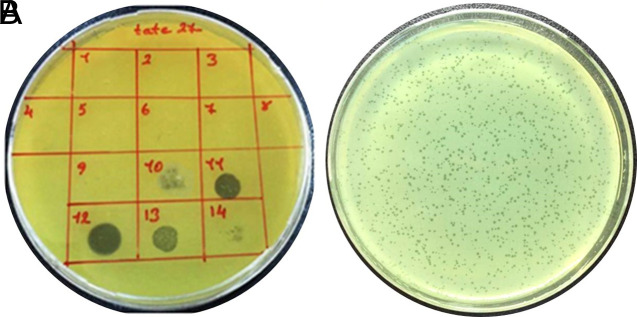
**(a)** Initial isolation of phages by the agar spot assay using *Pseudomonas aeruginosa* TRPA 28 as host. (1) Enas3, (2) Enas9, (3) Kleb11, (4) Acpi12, (5) Citroy14, (6) Citrof16, (7) Enteroc21, (8) Eshco22, (9) Achrox13, (10) Citrof19, (11) Psauro20, (12) Psauro27, (13) Psauro28, and (14) Kleb29. **(b)** The single plaques of phage Achrox13 infecting *Achromobacter xylosoxidans* TRAX 13.

A total of 68 distinct gram-negative colonies were isolated from two wastewater samples and used as potential hosts for the lytic phages. Among them, 14 strains belonging to eight species, designated as *Achromobacter xylosoxidans* TRAX 13, *Acinetobacter pittii* TRAP 12, *Citrobacter youngae* TRCY 14, *Citrobacter freundii* TRCF16, *C. freundii* TRCF 19, *Enterobacter asburiae* TREA 3, *E. asburiae* TREA 9, *Enterobacter cloacae* TRENC 21, *Escherichia coli* TRESC 22, *Klebsiella pneumoniae* TRKP 11, *K. pneumoniae* TRKP 29, *Pseudomonas aeruginosa* TRPA 20, *P. aeruginosa* TRPA 27, and *P. aeruginosa* TRPA 28 served as hosts for the initial isolation of 14 different phages (Figure 1). The phages with clear or turbid plaques with 0.5-2 mm in diameters were designated as Achrox13, Acpi12, Citroy14, Citrof16, Citrof19, Enas3, Enas9, Enteroc21, Escho22, Klab11, Kleb29, Psauro20, Psauro27, and Psauro28 (Table 1).

### Host Range of the Phages

The host range of 14 phages was determined by cross-infection of the 14 phage-susceptible strains. The phages Acpi12 and Enas3 showed the broadest host range by establishing successful lytic infection with three different species being different from their original hosts. The phages Enas9, Citroy14, and Escho22 showed lytic activity against only the hosts from which they were isolated. The other nine phages were able to infect 2-4 strains belonging to one or two species. *P. aeruginosa* TRPA 27 and *K. pneumoniae* TRKP 29 were the most phage-susceptible strains by showing susceptibility to five different phages (Table 1).

### Antibiotic Susceptibility of the Host Strains

The antibiotic susceptibility of the strains was determined based on the EUCAST^10^ guidelines (Table 2). Most of the strains were resistant to ampicillin and amoxicillin–clavulanic acid. *A. xylosoxidans* TRAX 13 was determined to be MDR. All of the host strains were susceptible to ceftriaxone, cefuroxime, ertapenem, imipenem, meropenem, and colistin.

### Morphology of Phages

**Figure 2.**
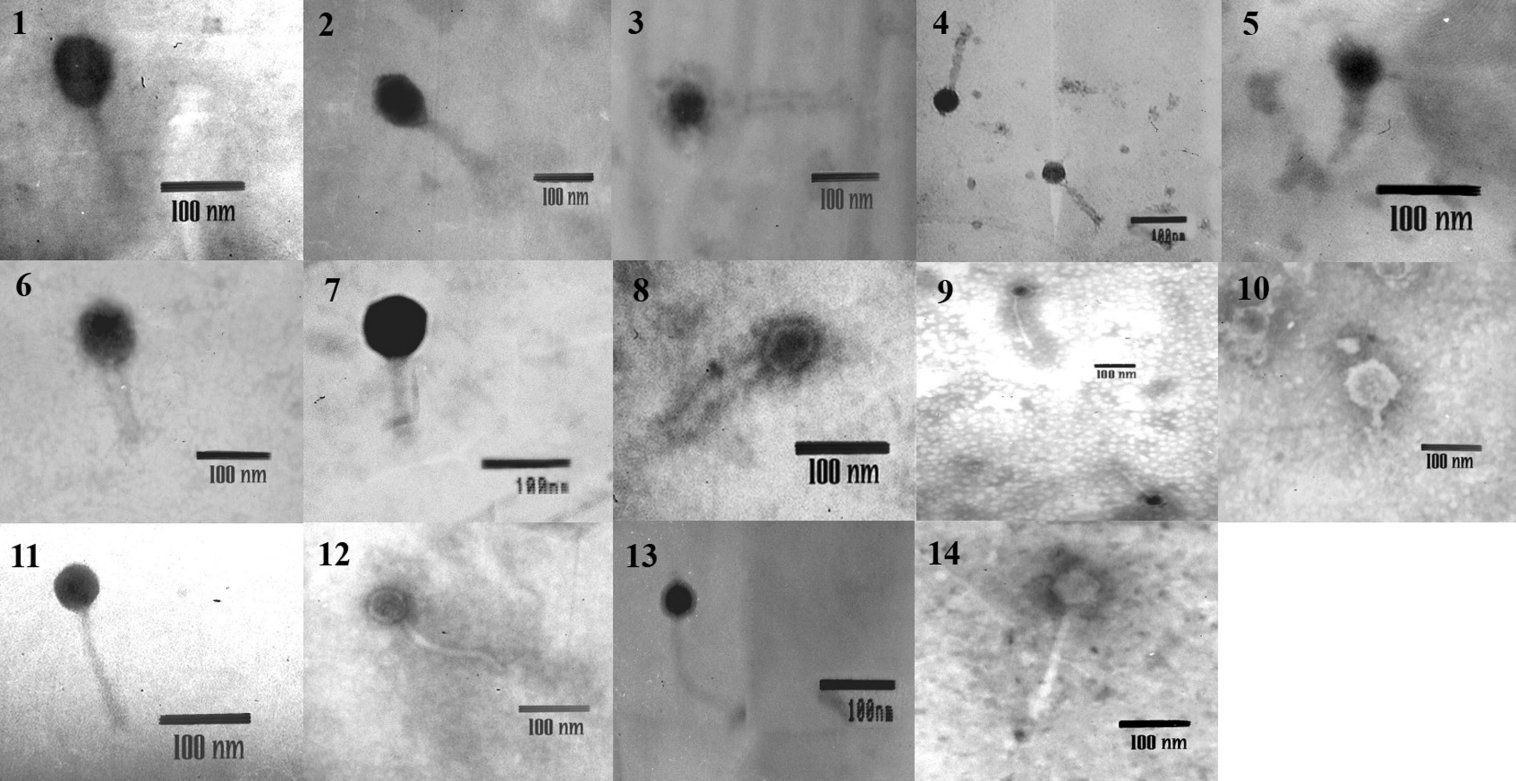
Transmission electron micrographs of phages. (1) Enas3, (2) Enas9, (3) Kleb11, (4) Acpi12, (5) Citroy14, (6) Citrof16, (7) Enteroc21, (8) Achrox13, (9) Eshco22, (10) Citrof19, (11) Psauro20, (12) Psauro28, (13) Psauro27, and (14) Kleb29.

Electron microscopy revealed that two phages, Citrof16 and Enteroc21, with icosahedral heads and contractile tails belonged to the *Myoviridae* family (Bradley classification A), 11 phages (Achrox13, Acpi12, Citroy14, Enas3, Enas9, Eshco22, Kleb11, Kleb29, Psauro20, Psauro27, and Psauro28) with icosahedral head and long, non-contractile tails belonged to the *Siphoviridae* family (Bradley classification B), and the phage Citrof19 with icosahedral head and short tail belonged to the *Podoviridae* family (Bradley classification C) (Figure 2). The head sizes of the phages varied from 36 × 45 nm to 100 × 117 nm and the tail lengths were from 31 to 228 nm (Table 3).

### The Burst Size of the Phage Enteroc21

**Figure 3.**
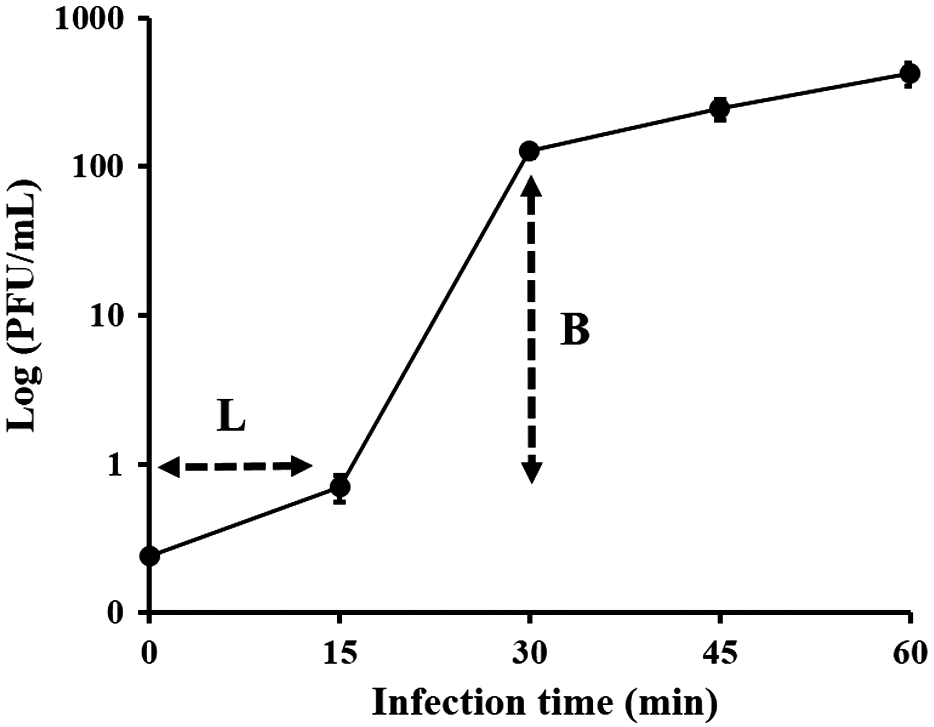
One-step growth curve of Enteroc21. L, latent period; B, burst period. Data are presented as the mean ± standard deviation.

The one-step growth experiment was used to determine the latent period and burst size of Enteroc21. The one-step growth curve was triphasic, including the latent, the log, and the stationary phases. The latent and the burst periods of Enteroc21 were estimated to be 15 minutes (Figure 3). The burst size of this phage was 180 per infected cell.

### Restriction Endonuclease Analysis of Phage Enteroc21 Genome

**Figure 4.**
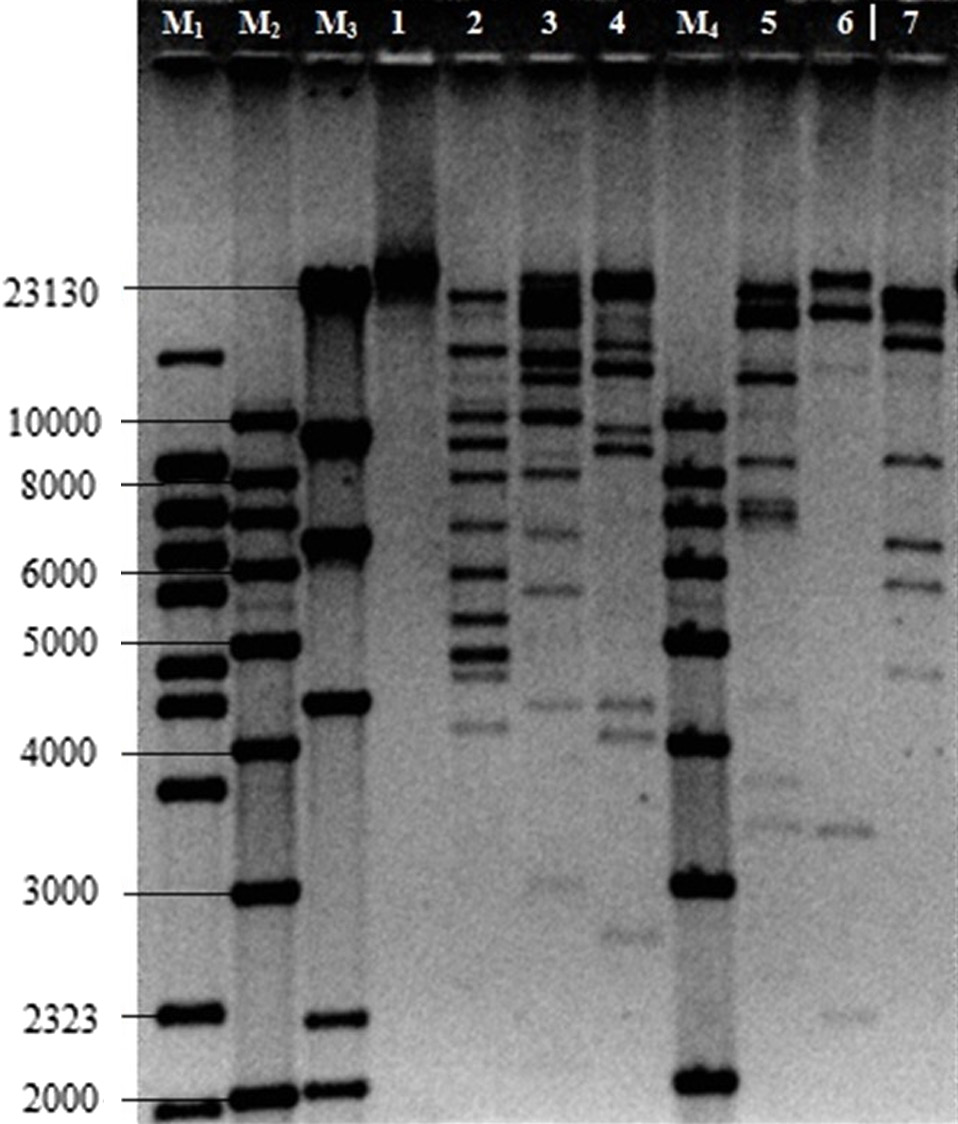
Restriction endonuclease digestion profiles of phage Enteroc21. M1, λ *BstE*II DNA marker; M2 and M4, GelPilot 1 kb DNA marker; M3, λ *Hind*III DNA marker. (1) Uncut phage DNA, (2) phage Enteroc21 *Hin*dIII, (3) *Ava*I, (4) *Pst*I, (5)* Eco*RI, (6)* Bst*EII, and (7) *Nco*I.

Several attempts were unsuccessful to digest the phage DNA isolated from the lysates of the phages generated from their hosts due to possible strong host restriction–modification or DNA modification system. To circumvent this problem, the phages were propagated in several methylation-deficient *E. coli* laboratory strains. Among the tested *E. coli* strains, Enteroc21 was able to be propagated in *E. coli* K-12 strain C600. Thus, the phage DNA was extracted from the lysate generated from this strain and digested with *Hin*dIII, *Ava*I, *Pst*I, *Eco*RI, *Bst*EII, and *Nco*I successfully. The genome of the Enteroc21 phage was estimated to be more than 100 kb based on the restriction endonuclease mapping (Figure 4).

## Discussion

The widespread increase of antibiotic resistance among pathogenic bacteria has brought the scientific world into the search for alternative antimicrobial agents to combat infections. One of these pursuits is the phage therapy that the scientific world has known for about a century. Although phage therapy has become unpopular after the discovery of antibiotics, studies on lytic phages have started to gain momentum in recent years with the emergence of new methods and technologies in microbiology.^15^ The presented study aimed to isolate lytic phages from wastewater with the potential to be used for phage therapy. Wastewaters are rich habitats from which lytic phages can readily be isolated with their respective host bacteria. The wastewaters are also good reservoirs for clinically important bacterial pathogens and their lytic phages.^16-18^ In this study, we isolated 14 lytic phages and determined their host range against eight gram-negative bacteria isolated from the same wastewater. Of these, 11 phages had a broad-host-range, which is a desirable feature of the lytic phages against which resistance will not develop in a short time in the phage therapy.^19^ The phages Acpi12 and Enas3 isolated from *A. pittii* TRAP 12 and *E. asburiae* TREA 3, respectively, showed the broadest host range with lytic activity against three different species other than their original hosts. To the best of our knowledge, this is the first report on a lytic phage propagated from *E. asburiae* with broad-host-range. Nevertheless, lytic phage, fHyAci03, and XC38 of *A. pittii* isolated from wastewaters have been shown to have the potential for use in phage therapy by infecting various clinical *Acinetobacter* strains.^20,21^ The phages Psauro20, Psauro27, and Psauro28 showed lytic activity against three different *P. aeruginosa* strains, and Pseuro20 and Pseuro28 had lytic activity against *K. pneumoniae* and *A. pittii* strains, respectively. The lytic phages of *Pseudomonas* strains with phage therapy potentials were reported by Jamal et al.^22^ They isolated a lytic phage designed as AZ1 from wastewater using *P. aeruginosa*-2995 strain and demonstrated its lytic activity against various clinical strains of *P. aeruginosa*, *A. xylosoxidans*, and *E. coli*.

Based on morphological features, the phages isolated in this study belong to the phage family of *Siphoviridae*, *Myoviridae*, and *Podoviridae*. These families from the order *Caudovirales* are the most abundant dsDNA phages in most habitats, and this has been shown in various studies from different environmental samples including wastewaters.^17,23,24^ Also, these families are widespread in nature that can be explained by their broad-host-range and the resistance of their protein capsids to environmental conditions.^25^ According to Bradley’s classification of phages, which groups phages based on their morphological characteristics,^26^ *Siphoviridae*, *Myoviridae*, and *Podoviridae* families are found in type A, B, and C, respectively. *Siphoviridae*, *Myoviridae*, and *Podoviridae* families are known to have a hexagonal head, and *Siphoviridae* members are distinguished from other phages by their non-contracting tails and their heads, which are usually 60 nm in diameter. The members of *Myoviridae* have a head and a tail separated by a neck. Their head can be between 50 and 110 nm in diameter, and the tail has a contractile sheath. The members of *Podoviridae* have a head that is about 60 nm in diameter and a short tail.^27^ Based on the said characteristics, it was determined that 11 phages belonged to *Siphoviridae*, two phages to *Myoviridae*, and one phage to *Podoviridae* family in the current study.

Genomic DNA of Enteroc21 was subjected to various restriction endonuclease analysis. In fact, it was aimed to cut and characterize the DNA of all phages using restriction endonucleases. Nevertheless, none of the phage DNA could be cut off since the DNA modification of the host cells might have been high. Then, various laboratory strains of *E. coli* were tried to be infected with 14 phages to solve this problem. As a result, only *E. coli* C600 was found to be susceptible to Enteroc21. This strain was then reinfected with Enteroc21 and high phage titration prepared for DNA isolation. The extracted DNA was cut using *Hin*dIII, *Ava*I, *Pst*I, *Eco*RI, *Bst*EII, and *Nco*I enzymes. The restriction fragments were compared using the DNA markers. This may be attributed to the point that *E. coli* C600 has the *mcrA*1 (mutation eliminating the restriction of DNA methylated at the sequence) gene. However, Kulikov et al.^27^ isolated 9 g phage from horse feces using *E. coli* C600 strain as the host. They tried to cut the phage’s DNA with the enzymes *Eco*RV, *Hae*III, *Dra*I, *Eco*RI, *Mbo*I, *Rsa*I, and *Ssp*I but were able to cut only with the enzymes *Rsa*I and *Ssp*I. Although there were cutting sites of the endonucleases in the phage genome based on sequence analysis, the failure of the cut process revealed that one or more of the DNA bases were modified. The genome size of the phage Enteroc21, which belonged to the *Myoviridae* family based on its electron microscopy, was estimated to be more than 100 kb based on restriction endonuclease mapping. The genomes of *Myoviridae* family phages have been reported to be linear, double-stranded DNA and ranging from 33 to 244 kb in size.^28^ The one-step growth curve gives knowledge about the life cycle of the virus in the host cell. The latent period is the interval it takes for a phage particle to reproduce within an infected host cell. The longer the latent phase duration, the larger the burst size of the phage is expected to be. The burst size gives the number of phage particles newly synthesized from an infected cell.^29^ The latent and the burst period of Enteroc21 were estimated to be about 15 minutes, and the burst size of Enteroc21 was 180 phages per infected cell. Many variations have been reported in the literature concerning latent time and burst size of phages. It has been reported that the latent period of phages belonging to the *Myoviridae* family, which were generally isolated from *Enterobacter* species, was between 11 and 20 minutes, and burst size was between 135 and 262 phages per infected cell.^30,31^ Our findings appear to be consistent with the literature, the phage Enteroc21 had a relatively high burst number with a short latent period. The main limitation of the study was that the lytic activities of the phages have not been determined against clinical isolates, which makes it difficult to estimate a true host range for these phages.

In conclusion, fourteen lytic phages were isolated and morphologically characterized in this study. One of the phages, Enteroc21, which had a broad-host-range, was further characterized for its potential to be used in phage therapy. Although phage therapy is not a new concept, further studies are needed to investigate the potential of these phages to be genetically identified and used in molecular biology, the food industry, and the treatment of bacterial diseases, including MDR strains.

## Figures and Tables

**Table 1.** Host Range of Phages

**Phage Code**	**Phage-Susceptible Bacteria**													
*Achromobacter xylosoxidans* TRAX 13	*Acinetobacter pittii* TRAP 12	*Citrobacter youngae* TRCY 14	*Citrobacter freundii* TRCF 16	*Citrobacter freundii* TRCF 19	*Enterobacter asburiae* TREA 3	*Enterobacter asburiae* TREA 9	*Enterobacter cloacae* TRENC 21	*Escherichia coli* TRESC 22	*Klebsiella pneumoniae* TRKP 11	*Klebsiella pneumoniae* TRKP 29	*Pseudomonas aeruginosa* TRPA 20	*Pseudomonas aeruginosa* TRPA 27	*Pseudomonas aeruginosa* TRPA 28
Achrox13	+*	−	−	−	−	−	−	−	−	−	−	−	−	**+**
Acpi12	−	+*	−	**+**	−	−	−	−	**+**	−	**+**	−	−	−
Citroy14	−	−	+*	−	−	−	−	−	−	−	−	−	−	−
Citrof16	−	−	−	+*	**+**	−	−	−	−	−	**+**	−	−	−
Citrof19	−	−	−	−	+*	−	−	−	−	−	−	−	**+**	−
Enas3	−	**+**	−	−	−	+*	−	**+**	−	−	**+**	−	−	−
Enas9	−	−	−	−	−	−	+*	−	−	−	−	−	−	−
Enteroc21	−	−	−	−	−	−	**+**	+*	−	−	−	−	−	−
Eshco22	−	−	−	−	−	−	−	−	+*	−	−	−	−	−
Kleb11	−	**+**	−	−	−	−	−	−	−	+*	−	−	−	−
Kleb29	−	−	−	−	−	−	−	−	−	−	+*	−	**+**	−
Psauro20	−	−	−	−	−	−	−	−	−	−	**+**	+*	**+**	**+**
Psauro27	−	−	−	−	−	−	−	−	−	−	−	**+**	+*	**+**
Psauro28	−	**+**	−	−	−	−	−	−	−	−	−	**+**	**+**	+*

+, the lytic activity; −, no lytic activity; *, original host from which phage was isolated.

**Table 2.** Antibiotic Susceptibility Profiles of Phages-Susceptible Bacteria

**Strain Name**	**Antibiotics**																	
**Ampicillin**	**Piperacillin**	**Cefepime**	**Ceftazidime**	**Ceftriaxone**	**Cefuroxime**	**Amoxicillin–clavulanic acid**	**Piperacillin–tazobactam**	**Aztreonam**	**Ertapenem**	**Imipenem**	**Meropenem**	**Amikacin**	**Gentamicin**	**Netilmicin**	**Ciprofloxacin**	**Colistin**	**Trimethoprim–sulfamethoxazole**
*Enterobacter asburiae* TREA 3	**R**	S	S	S	S	-	**R**	S	S	S	S	S	S	S	S	S	S	S
*Enterobacter asburiae* TREA 9	**R**	**R**	S	S	S	-	**R**	S	S	S	S	S	S	S	I	S	S	S
*Klebsiella pneumoniae* TRKP 11	**R**	S	S	S	S	S	S	S	S	S	S	S	S	S	S	S	S	S
*Acinetobacter pittii* TRAP 12	-	-	S	I	-	-	-	S	-	-	S	S	S	S	S	S	S	-
*Citrobacter youngae* TRCY 14	**R**	S	S	S	S	-	**R**	S	S	S	S	S	S	S	S	S	S	S
*Citrobacter freundii* TRCF 16	**R**	S	S	S	S	-	**R**	S	S	S	S	S	S	S	S	S	S	S
*Enterobacter cloacae* TRENC 21	**R**	S	S	S	S	-	**R**	S	S	S	S	S	S	S	S	S	S	S
*Escherichia coli* TRESC 22	**R**	**R**	S	S	S	S	**R**	**R**	S	S	S	S	S	S	S	S	S	**R**
*Achromobacter xylosoxidans* TRAX 13	-	S	**R**	S	-	-	-	S	**R**	-	S	S	I	**R**	**R**	**R**	S	-
*Citrobacter freundii* TRCF 19	**R**	**R**	S	S	S	-	**R**	S	S	S	S	S	S	S	S	S	S	S
*Pseudomonas aeruginosa* TRPA 20	-	S	S	S	-	-	-	S	I	-	S	S	S	S	S	S	S	-
*Pseudomonas aeruginosa* TRPA 27	-	S	S	S	-	-	-	S	I	-	S	S	S	S	S	S	S	-
*Pseudomonas aeruginosa* TRPA 28	-	S	S	S	-	-	-	S	I	-	S	S	S	S	S	S	S	-
*Klebsiella pneumoniae* TRKP 29	**R**	S	S	S	S	S	S	S	S	S	S	S	S	S	S	S	S	S

S, susceptible; R, resistant; I, intermediate susceptible; -, not tested.

**Table 3.** General Characteristics of Phages

Phages	Family	Bradley Classification	Head Diameter (nm)	Tail Length (nm)	Plaque Appearance	Plaque Diameter (mm)
Citrof16	*Myoviridae*	A	69 × 100	100	Clear	2
Enteroc21	*Myoviridae*	A	100 × 117	133	Clear	1-2
Enas3	*Siphoviridae*	B	59 × 118	112	Clear	<1
Enas9	*Siphoviridae*	B	67 × 100	73	Clear	<1
Kleb11	*Siphoviridae*	B	53 × 53	170	Clear	<1
Acpi12	*Siphoviridae*	B	36 × 45	118	Turbid	1
Citroy14	*Siphoviridae*	B	43 × 43	76	Clear	2
Eshco22	*Siphoviridae*	B	57 × 57	228	Turbid	2
Achrox13	*Siphoviridae*	B	50 × 50	95	Turbid	<1
Psauro20	*Siphoviridae*	B	50 × 50	128	Clear	1
Psauro27	*Siphoviridae*	B	50 × 50	168	Clear	2
Psauro28	*Siphoviridae*	B	58 × 64	186	Clear	<1
Kleb29	*Siphoviridae*	B	69 × 69	223	Turbid	1
Citrof19	*Podoviridae*	C	92 × 84	31	Turbid	<1
